# A Case of Irido-Choroiditis

**Published:** 1884-01

**Authors:** 

**Affiliations:** Vienna Vet-institute


A CASE OF IRIDOCHORIOIDITIS "WALL EYES."—By Prof. Dr. Bayer, of Vienna Vet-institute. The patient was brought to the clinic on account of hoof disease but we at once observed a peculiar intensive yellow coloring of the iris of both eyes, even at a distance.

There seemed to be no evidence of photophobia in either eye; an accumulation of mucous was present in the inner canthus of each eye; the lids were slightly swollen; the conjunctiva and sclerotica very much injected; the cornea clear; the horizontal diameters of the anterior chamber appeared to be less than normal, the aqueous humour clear.

The changes upon the iris were however very striking. Instead of the usual whitish color normal to such eyes it had a sulphur-yellow, or better, a pus yellow color; the dark shades which are generally seen in such eyes were not visible. The superior part of the iris appeared to be distended as a sort of sack. Vessels, having the diameter of *horse-hairs,* were to be seen taking a regular and at other places a serpentine course from the peripheries of the iris towards the centre, some of which continued to the wart like projections that extend over the pupil; the vessels were so distended as to be above the level of the iris proper. The edges of the pupil were in such intimate opposition that no view could be had of the interior of the eye with the opthalmoscope. The eye-ball did not seem to possess any increase of tenderness or density upon palpation. I concluded to apply atropia solution the right eye and to leave the other untouched.

No changes in the conditions were discernable on the next day except that the right eye was more photophobic and a greater secretion of mucus had taken place. The atropia had not had any effect upon the iris; the posterior surface of the cornea had become coated in its inferior parts; this condition had increased on the third day and the aqueous humour had also become clouded in its inferior portion; the superior half of the iris, in both eyes, had assumed a more whitish yellow color. The previously mentioned blood vessels had, in the right eye, become somewhat less distended, of a brown instead of a bright red color, and at times their continuity had become indistinct, which gave to the iris a peculiar appearance as if covered with brown spots connected with very delicate threads of the same color. Quite a large extravasation of blood had taken place in the extreme peripherie of the iris. The pupil remained contracted.

No changes had occured upon the vessels of the iris of the left eye.

On the fourth day the superior parts of the iris were seen to be still more pale. The dots, previously mentioned in the iris of the right eye had almost entirely disappeard. The extravasation had become separated into smaller portions from absorption. In hæmorrhagic exudation was apparent in the inferior part of the anterior chamber of the *left eye*, and an extravasation similar to that of the right eye had appeared. A whitish exudation was to be seen projecting over the edges of the pupil; the vasculazation of the iris was very indistinct; the photophobia was much increased and the patient very shy of being handled about the eyes.

The improvement in the right eye continued; the iris assuming its normal color and the pupil becoming somewhat distended, but no view could be obtained of the interior of the eye on account of the clouded condition of the cornea.

The left eye, however, did not improve the disease progressing in a marked degree; the anterior chamber becoming filled with a hæmorrhagic exudation, which appeared to have coagulated, its superior portion being of a bright red color, while in the lower, numerous irregular formed dark red masses could be seen. This condition inproved under treatment with warm applications, but the horse was removed from the clinic so that I could not study the change further.

